# Long non-coding RNA ATB promotes glioma malignancy by negatively regulating miR-200a

**DOI:** 10.1186/s13046-016-0367-2

**Published:** 2016-06-06

**Authors:** Chun-Chun Ma, Zhang Xiong, Guan-Nan Zhu, Chao Wang, Gang Zong, Hong-Liang Wang, Er-Bao Bian, Bing Zhao

**Affiliations:** Department of Neurosurgery, The Second Affiliated Hospital of Anhui Medical University, 678 Fu Rong Road, Hefei, Anhui Province China 230601; Cerebral Vascular Disease Research Center, Anhui Medical University, 678 Fu Rong Road, Hefei, Anhui Province China 230601

**Keywords:** Glioma, lncRNA, ATB, miRNA, miR-200a, ceRNA, TGF-β2

## Abstract

**Background:**

Glioma is one of the most common and aggressive primary malignant tumor in the brain. Accumulating evidences indicated that aberrantly expressed non-coding RNAs (ncRNAs), including long non-coding RNAs (lncRNAs) and microRNAs (miRNAs), contribute to tumorigenesis. However, potential mechanisms between lncRNAs and miRNAs in glioma remain largely unknown.

**Methods:**

Long non-coding RNA activated by TGF-β (LncRNA-ATB) expression in glioma tissues and cells was quantified by quantitative reverse transcription–PCR. Glioma cell lines U251 and A172 were transfected with sh-ATB, miR-200a mimics, miR-200a inhibitors, after we assayed the cell phenotype and expression of the relevant molecules. Dual-luciferase reporter assay, RIP and a xenograft mouse model were used to examine the expression of sh-ATB and its target gene miR-200a.

**Results:**

ATB is abnormally up-regulated both in glioma tissues and cell lines compared with normal brain tissues, and glioma patients with high ATB expression had shorter overall survival time. Knockdown of ATB significantly inhibits glioma malignancy, including cell proliferation, colony formation, migration, invasion in vitro, and the xenograft tumor formation in vivo. In addition, ATB was confirmed to target miR-200a, and miR-200a inhibition reversed the malignant characteristics of ATB knockdown on glioma cells. In particular, ATB may act as a ceRNA, effectively becoming a sink for miR-200a, thereby modulating the derepression of TGF-β2.

**Conclusions:**

Our findings suggest that ATB plays an oncogenic role of glioma cells by inhibiting miR-200a and facilitating TGF-β2 in glioma, thereby may represent a potential therapeutic target for the treatment of human glioma.

## Background

Glioma poses a tremendous threat to public health with an incidence of ~5 cases per 100,000 persons and its mortality is high around the world. Glioma is categorized into four histopathologic grades (I–IV) based on the degree of malignancy according to World Health Organization (WHO) classification [1]. The majority patients suffered from glioma are diagnosed at the advanced stages and exhibit extremely poor prognosis. Combined with maximal safe surgical resection, standard radiation therapy and chemotherapy, the most advanced treatment is formed, while the most malignant glioma called gliobastoma multiform is lined with an average life expectancy of only 14 months [2]. Fortunately, significant progress has been made in understanding the molecular mechanisms of glioma. Though clinical application is largely palliative, the fatality rate remains high in glioma patients. Therefore, it is extremely vital for us to elucidate new mechanisms associated with glioma development and establish potential therapeutic targets for the treatment of human glioma.

Just as the human genome project which delineated that only a small amount of mammalian genome is encoded proteins while the vast majority of mammalian genome are transcribed as non-coding RNAs (ncRNAs), such as long noncoding RNAs(lncRNAs) [3, 4]. LncRNAs are a class of transcripts longer than 200 nucleotides in length with little functional protein-coding ability. Recently, many studies have demonstrated that lncRNAs could regulate gene expression at multiple levels, including transcriptional, post-transcriptional and epigenetic modulation [5–7]. Numerous studies also indicated that dysregulated lncRNAs are involved in the biological process during cancer development and progression [8, 9] Examples like lncRNAs include HOTAIR (HOX transcript antisense intergenic RNA) [10], CRNDE(Colorectal neoplasia differentially expressed) [11], MEG3 (Maternally Expressed Gene 3) [12]. Yet, the biological roles of lncRNAs in glioma are largely unknown. Long noncoding RNA activated by TGF-β (lncRNA-ATB) was initially identified as a lncRNA in Hepatocellular carcinoma (HCC) and its expression was significantly up-regulated both in HCC cells and HCC samples [13]. Furthermore, ATB was also overexpressed in other cancers, including breast cancer [14] and colorectal cancer [15]. Therefore, the aberrant expression level of ATB was involved in a series of cancer progression. However, functional mechanism and potential biological role of ATB in human glioma are still unknown.

MiRNAs are highly conserved among species, and play important roles in a variety of biological and pathological processes. A number of studies indicated that deviant expression of miRNAs contributes to tumorigenesis and plays a critical role in regulating the biological behaviors of tumor cells by modulating the protein or mRNA levels of its downstream target genes [16, 17]. Dysregulation of miRNAs in glioma has also been reported, and certain miRNAs have been functionally involved in glioma. Previous studies have demonstrated that miR-200a as a member of the miR-200 family, which exerts as a tumor-suppressor gene and is down-regulated in many tumors, including glioma [18]. However, the molecular mechanism of miR-200a deregulation and how such deregulation contributes to glioma tumorigenesis remains abstruse.

In the present study, we aimed to investigate the functional expression and clinical significance of ATB in human glioma. ATB was significantly up-regulated both in glioma tissues and cell lines, whereas knockdown of ATB diminished cell proliferation, migration and invasion in glioma. In addition, the interaction among ATB, miR-200a and TGF-β2 was also studied in order to reveal the underlying mechanisms. We identified that ATB may act as a ceRNA of miR-200a, which resulted in the derepression of TGF-β2. These findings will give a novel strategy for the treatment of glioma.

## Methods

### Human tissue samples

Seventy-nine glioma tissues and 15 normal brain tissues (NBTs) were obtained from the Department of Neurosurgery, the Second Affiliated Hospital of AnHui Medical University during 2011–2014. These glioma samples were from 50 males and 29 females with age ranging from 13 to 73 years (median, 47 years). All samples had confirmed pathological diagnosis and were divided into low grade (grade I–II) and high grade (grade III–IV) according to the WHO classification by neuropathologists. Informed consents were obtained from all patients, and this study was approved by the Clinical Research Ethics Committee at the Second Affiliated Hospital of AnHui Medical University.

### Cell culture

The human glioma cell lines (U251 and A172) were purchased from the Chinese Academy of Sciences (Shanghai, China) and cultivated in Dulbecco’s modified Eagle’s medium (DMEM; Hyclone, Logan, UT, USA) with high glucose supplemented with 10 % fetal bovine serum (FBS, Gibco, Carlsbad, CA, USA) and streptomycin (100 μg/ml), penicillin (100 U/ml). All cell lines were cultured at 37 °C in a humidified incubator with 5 % CO_2_.

### Cell transfection

Short-hairpin RNA plasmid directed knock-down human ATBs (GenePharma, Shanghai, China), and was indicated as sh-ATB (sh-ATB sense:5'-GATCCGCCTGTCTGTATTTGCGAATACCTTTTTCAAGAGAAAAGGTATTCGCAAATACAGACAGGCTTTTTTG-3' and anti-sense: 5'-AATTCAAAAAAGCCTGTCTGTATTTGCGAATACCTTTTCTCTTGAAAAAGGTATTCGCAAATACAGACAGGCG-3') and its corresponding non-targeting sequence (sh-control) (sh-control sense: 5'-GATCCGTTCTCCGAACGTGTCACGTTTCAAGAGAACGTGACACGTTCGGAGAACTTTTTTG-3' and anti-sense: 5'-AATTCAAAAAAGTTCTCCGAACGTGTCACGTTCTCTTGA AACGTGACACGTTCGGAGAACG-3') plasmid (GenePharma, Shanghai, China) were transfected into U251 and A172 cells respectively by using Lipofectamine2000 (Invitrogen, USA) according to the manufacturer’s protocol. MiR-200a mimics, miR-200a inhibitors and miR-200a negative control (NC) were obtained from RiboBio (RiboBio, Guangzhou, China), and transfected into cell lines as the above described.

### RNA extraction and quantitative Real-time PCR

Total RNAs were extracted from tissues and cultured cells using Trizol reagent (Invitrogen, USA) according to the manufacturer’s protocol. Using a Nanodrop Spectrophotometer (IMPLEN GmbH, Munich, Germany), RNA concentration and quality were determined by the 260/280 nm absorbance. Then RNA was reversely transcribed into High Capacity cDNA by using PrimeScript™ RT Master Mix (Perfect Real Time) (TaKaRa Biotechnology, Dalian, China). Maxima SYBR Green/ROX qPCR Master Mix (Thermo Fisher Scientific, USA) was used for quantitative Real-time PCR. The primers for genes were determined as follows: ATB forward 5′-ACAAGCTGTGCAGTCTCAGG-3′, reverse 5′- CTAGGCCCAAAGACAATGGA-3′; TGF-β2 forward 5′-CACCATAAAGACAGGAACCTG-3′, reverse 5′-GGAGGTGCCATCAATACCTGC-3′; GAPDH forward 5′-AGCAAGAGCACAAGAGGAAG-3′, reverse 5′-GGTTGAGCACAGGGTACTTT-3′. All-in-One™ miRNA First-Strand cDNA Synthesis Kit (Genecopoeia, Guangzhou, China) was used for miRNA reverse transcription and RT-QPCR was conducted using All-in-One™ miRNA qPCR Kit (Genecopoeia, Guangzhou, China) of miR-200a and U6 (miR-200a (Cat#HmiRQP0298) and U6 (Cat#HmiRQP9001), Genecopoeia, Guangzhou, China), respectively, by using the ABI 7100 (Applied Biosystems, Darmstadt, Germany). GAPDH and U6 were used as internal controls for ATB, TGF-β2 and miR-200a. PCR Cycling conditions for mRNA was 2 min at 50 °C, 10 min at 95 °C and followed by 45 cycles of 95 °C for 15 s, 60 °C for 60s; for miRNA was 10 min at 95 °C and followed by 45 cycles of 95 °C for 10s, 60 °C for 20s and 72 °C for 12 s. All qRT-PCR reactions were performed in triplicate. Relative quantification of gene expression was calculated by the 2^−ΔΔCt^ method.

### Cell proliferation assay

Cell Counting Kit-8 (CCK8, Beyotime Institute of Biotechnology, Jiangsu, China) was used to detect the cell^,^s proliferation ability. Cells were placed into 96-well plates at the density of 2000 cells/well. Approximately 20 μl of CCK8 regent was added to each well after transfection, and incubated at 37 °C for 2 h. Absorbance was measured at a wavelength of 450 nm by using a ST-360 micro-plate reader (KHB, Shanghai, China). Three replicate wells were set up in each group and experiments were repeated three times.

### Clony formation assay

For the clone formation assay, approximately 200 viable cells were seeded in 6-well plates in triplicate and incubated at 37 °C with 10 % fetal bovine serum. After 12 days, cells were fixed with 4 % polyoxymethylene and stained with 1.5 % methylene blue. Colony formation ratio was calculated as (number of cells/initiative cell × 100 (%)).

### Wound healing assay

Cells were cultured in 6-well plates. When 95 % confluency was reached, cell layers were wounded using a 10 μL tip to produce a gap, gently washed, and cultured with serum-free medium for 24 h. The wounded gaps were photographed with a light microscope (IX71; Olympus, Tokyo, Japan) at × 200 magnification. Lines were drawn along the leading edges of the cells, and the gap distances of migrating cells from five different areas for each wound were measured and analyzed.

### Cell invasion assays

Cell invasion ability was tested by using 24-well chambers with 8 μm pore size (Corning, USA). 8 × 10^4^ cells were resuspended in 150 μl serum-free medium and seeded into the upper chamber pre-coated with 500 ng/ml Matrigel solution (BD, USA) invasion assay, while 500 μl of 10 % FBS medium was placed in the lower chamber, After incubation at 37 °C for 48 h for invasion assay. Cells on the upper chamber membrane were scraped off by cotton swab. Cells on the lower chamber membrane were fixed with 4 % polyoxymethylene and stained with 0.1 % crystal violet. Five predetermined fields were counted under a microscope (×200). All assays were performed in triplicate.

### Luciferase reporter assays

ATB fragment containing the predicted miR-200a binding site, the putative sequences of the binding site then cloned into a pmirGlO Dual-luciferase miRNA Target Expression Vector (Promega, Madison, WI, USA) to form the reporter vector pmiRGLO-ATB-wild-type (ATB-WT). To mutate the putative binding site of miR-200a in ATB gene, the sequence of putative binding site was replaced as indicated and was named as pmiRGLO-ATB-mutated-type (ATB-MUT). pmirGLO-ATB-WT or pmirGLO-ATB-MUT was cotransfected with miR-200a mimics or miR-200a NC into glioma cells by using Lipofectamie 2000 (Invitrogen, USA). After 48 h transfection for luciferase assay using a Dual-Luciferase Reporter Assay System (Promega, Madison, WI, USA) according to the manufacturer’s protocol.

Similarly the 3’-UTR of TGF-β2 containing the putative miR-200a binding site, the putative sequences of the binding site were cloned into a pmirGlo Dual-luciferase miRNA Target Expression Vector to form the reporter vector TGF-β2-wild-type (TGF-β2-WT) (GenePharma). To mutate the putative binding site of miR-200a in the 3′-UTR-containing vector, the sequence of putative binding site was replaced as indicated and was named as TGF-β2-mutated-type (TGF-β2-MUT). The transfection procedure and measurement of Luciferase activities were handled similarly as described above. All assays were independently performed in triplicate.

### RNA immunoprecipitation

EZ-Magna RIP RNA-binding protein immunoprecipitation kit (Millipore, Billerica, MA, USA) was used in RNA immunoprecipitation (RIP). RIP was implemented to pull down endogenous miR-200a associated with ATB in glioma cell lines, and was performed following the manufacturer's protocol. U251 and A172 cells were lysed by RIP lysis buffer, 100 μL of cell lysate was incubated with RIP immunoprecipitation buffer containing magnetic beads conjugated with human anti-Argonaute2 (Ago2) antibody (Millipore) and normal mouse IgG (Millipore) was indicated as negative control. Samples were incubated with Proteinase K buffer and then target RNA was extracted. Purified RNA was subjected to RT-QPCR analysis for further study.

### Western blot analysis

Total proteins were extracted from the cells using RIPA buffer with PMSF (Beyotime Institute of Biotechnology) on ice, subjected to SDS-PAGE and electrophoretically transferred to polyvinylidene difluoride (PVDF) membranes. Membranes were incubated in 5 % nonfat milk dissolved in Tris-buffered saline (TBS) containing 0.1 % Tween-20 for 1.5 h at room temperature and then incubated with primary antibodies as follows: TGF-β2 (1:1000, Abcam, EUGENE, USA), β-actin (1:1000, Santa Cruz Biotechnology). After incubation with secondary antibodies (Goat anti-rabbit or Goat anti-mouse, 1:5000 respectively; ZSGB-BIO, Beijing, China), immune complexes were visualized by SuperSignal® West Femto Trial Kit (Thermo Fisher, USA) and blot bands were scanned using Find-do × 6 Tanon (Tanon, Shanghai, China).

### Tumor xenograft formation assay in nude mice

Four-week-old female BALB/C athymic nude mice were purchased from the National Laboratory Animal Center (Beijing, China). Experiments with nude mice were conducted strictly in accordance with a protocol approved by the Administrative Panel on Laboratory Animal Care of the Second Affiliated Hospital of AnHui Medical University. The animals were free to autoclaved food and water during the study. U251 cells transfected with sh-control or sh-ATB were collected, and 3 × 10^6^ cells were subcutaneously injected into the left flank of the nude mice. Tumor volumes were examined every 5 days, and after 40 days, the mice were killed and tumor tissues were excised and weighed for further study.

### Immunohistochemistry

Tumor tissues from subcutaneous implantation assay were fixed in 4 % paraformaldehyde, and then were dehydrated, embedded in paraffin, and cut. Consecutive 4 μm thick sections were analyzed by immunohistochemistry using antibodies against Ki-67 (Santa Cruz Biotechnology, USA).

### Statistical analysis

Experimental data were presented as means ± standard deviation (SD). GraphPad Prism V5.0 (GraphPad Software, Inc., La Jolla, CA, USA) software was used for statistical analysis. Differences were analyzed by SPSS 17.0 statistical software with the Student’s t-test or one-way ANOVA. The relationship between the expression of ATB, miR-200a and TGF-β2 in tissues was analyzed with Pearson’s correlation. Survival analysis was performed using the log-rank test in GraphPad Prism 5. Differences were considered significant if *P* < 0.05. Corresponding significance levels are indicated in the figures.

## Results

### High expression of ATB was correlated with poor outcome of glioma patients

**Fig. 1:**
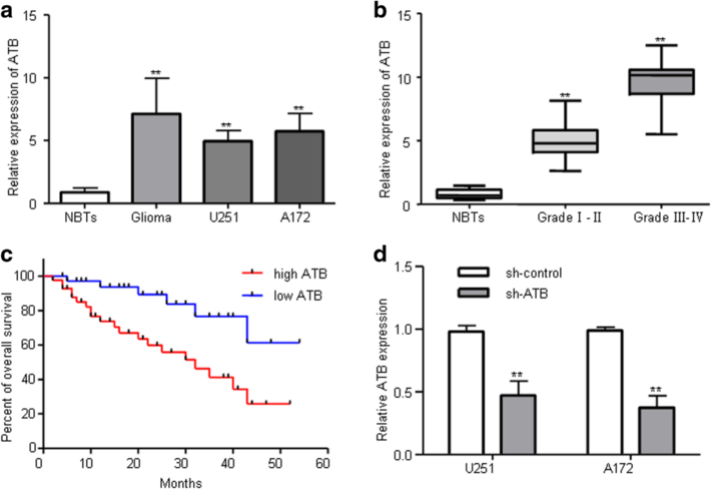
ATB was highly expressed in human glioma. **a** RT-QPCR analysis of ATB expression in normal brain tissues (NBTs) (*n* = 19), glioma tumor tissues (*n* = 79) and glioma cell lines (U251 and A172). **P < 0.01 vs. NBTs group. **b** Expression levels of ATB in glioma tissues of positively correlated with tumor grades. **P < 0.01 vs. NBTs group. **c** Kaplan-Meier analyses of the associations between ATB expression level and overall survival of patients with human glioma (The log-rank test was used to calculate *P*-values). **P < 0.01 vs. low ATB expression group. **d** Relative expression levels of ATB after glioma cells transfected with sh-ATB and sh-control. **P < 0.01 vs. sh-control group

To define the role of ATB in glioma, using real-time quantitative PCR (RT–QPCR) analysis, we measured ATB expression levels in 79 glioma tissues, 19 normal brain tissues and two glioma cell lines (U251 and A172). ATB expression levels in glioma tissues and cell lines were significantly increased compared with normal brain tissues (Fig. 1a). To determine whether ATB expression was associated with the grade malignancy in gliomas, we examine mRNA expression of ATB. We showed that ATB expression was positively correlated with the pathological grades of glioma (Fig. 1b). We then further assess whether the expression of ATB is correlated with the postoperative survival time of human glioma. The Kaplan–Meier method and log-rank test revealed that high ATB expression level was inversely correlated with glioma patients’ overall survival (Fig. 1c).

### Knockdown of ATB inhibited glioma cells proliferation, migration, invasion in vitro

**Fig. 2:**
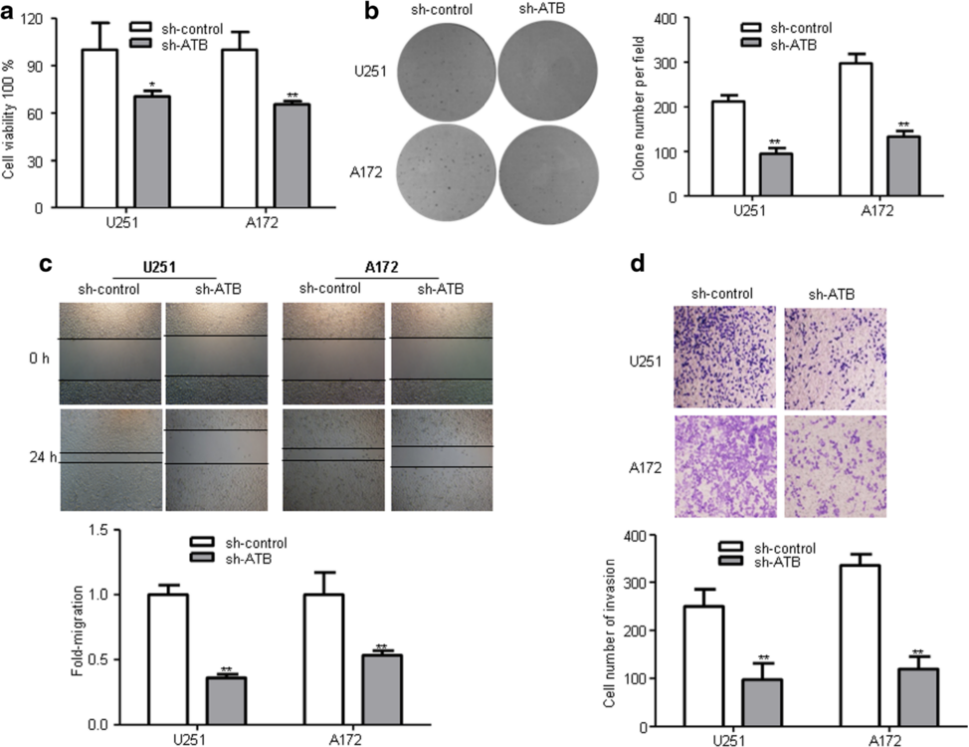
Knockdown of ATB inhibited the proliferation, migration, and invasion of glioma cells in vitro. **a** CCK-8 assay was performed to determine the proliferation effect of sh-ATB and sh-control transfected U251 and A172 cells. *P < 0.05, **P < 0.01 vs. sh-control group. **b** Clony formation assay was performed to detect the proliferation of U251 and A172 cells after transfected with sh-ATB and sh-control. **P < 0.01 vs. sh-control group. **c** Wound healing assay to evaluate the effect of ATB on cell migration in U251 and A172 cells. **P < 0.01 < 0.05 vs. sh-control group. **d** Invasion assay (use matrigel transwell chambers) for investigating cell invasiveness. **P < 0.01 vs. sh-control group. Data are presented as mean ± SD from three independent experiments

To explore the biological functions of ATB in glioma, we firstly designed an ATB knockdown model using transfection of sh-ATB plasmid in U251 and A172 glioma cell lines and its knockdown efficiency was remarkable compared with sh-control (Fig. 1d). CCK-8 assays were performed to detect the impact of ATB knockdown on cell viability of glioma cell lines (U251 and A172). We found that ATB knockdown inhibited cell viability compared to that of cells transfected with sh-control group (Fig. 2a). Furthermore, colony formation assay showed that ATB knockdown exhibited remarkable decreased clone numbers and colony size compared with sh-control group both in U251 and A172 glioma cells (Fig. 2b). To further explore the effect of ATB on glioma cells migration and invasion. Wound healing assay showed that knockdown of ATB impeded the migration in monolayer cultured U251 and A172 glioma cells (Fig. 2c). In addition, numbers of invaded cells were obviously attenuated in the sh-ATB groups compared with sh-control groups (Fig. 2d). Therefore, these results indicated that ATB might act as an oncogene in regulating glioma biological process.

### ATB directly targeted miR-200a and inhibited its expression in glioma cells

Previous studies have demonstrated that lncRNAs could function as a competing endogenous RNAs (ceRNA) by competitively binding miRNAs, such as miR-200a, in hepatocellular carcinoma [8, 13, 19].

**Fig. 3:**
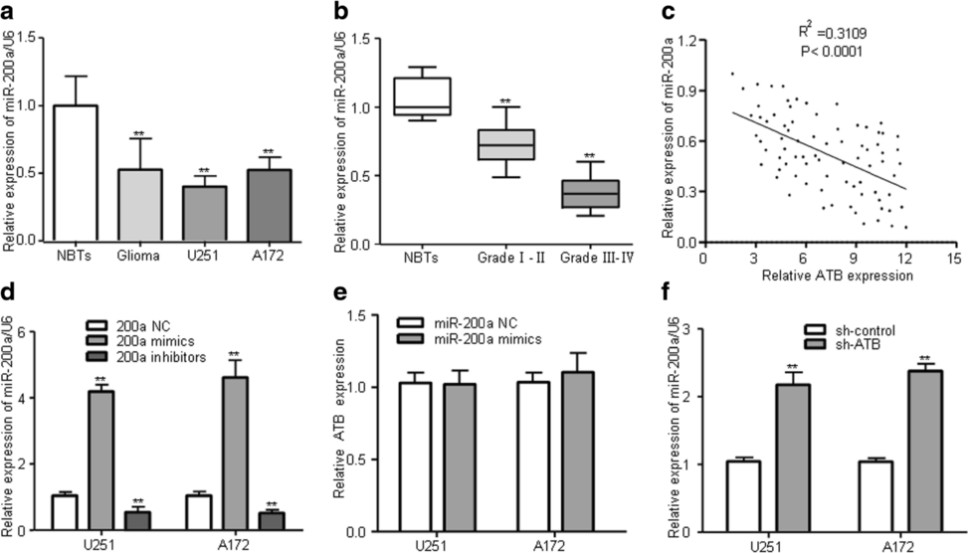
miR-200a expression was inversely associated with ATB expression. **a** MiR-200a was lowly expressed in glioma tissues and cell lines. **P < 0.01 vs. NBTs group. **b** miR-200a expressed levels was inversely correlated with pathological grades of glioma. **P < 0.01 vs. NBTs group. **c** Pearson’s correlation analysis of the relationship between ATB expression and miR-200a level in 79 human glioma patients. **d** The efficiency of miR-200a expression levels after glioma cells transfected with miR-200a NC, miR-200a mimics and miR-200a inhibitors. **P < 0.01 vs. miR-200a NC group. **e** Relative expression of ATB in U251 and A172 cells transfected with miR-200a mimics and miR-200a NC. **f** Relative expression of miR-200a in U251 and A172 cells transfected with sh-ATB and sh-control. **P < 0.01 vs. sh-control group. Data were presented as mean ± SD from three independent experiments

Thus we intended to investigate the potential interaction between ATB and miR-200a in human glioma. As shown Fig. 3a, using QRT-PCR analysis, we found that the expression of miR-200a was lowly expressed in glioma tissues compared with normal brain tissues (NBTs). We further found that miR-200a level in glioma tissues was inversely correlated with pathological grades of glioma (Fig. 3b). We further found that the expression level of ATB was inversely correlated with the expression of miR-200a in 79 human glioma patients (Fig. 3c).

Then, U251 and A172 glioma cells were transfected with miR-200a NC, miR-200a mimics and miR-200a inhibitors (Fig. 3d). To further elucidate whether ATB was regulated by miR-200a, we compared ATB expression levels in U251 and A172 cells transfected with miR-200a NC or miR-200a mimics and the results showed that overexpression of miR-200a did not reduce the expression of ATB in glioma cells (Fig. 3e). However, knockdown of ATB significantly increased the expression levels of miR-200a compared to the control group (Fig. 3f).

**Fig. 4:**
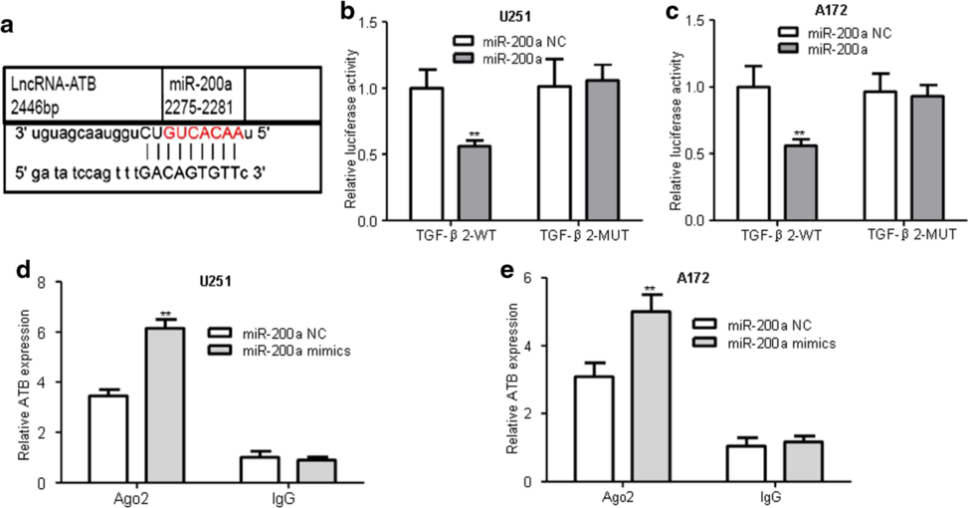
ATB directly targeted miR-200a in glioma. **a** The predicted miR-200a binding sites on ATB. **b**-**c** Luciferase activity in U251 and A172 glioma cells co-transfected with miR-200a mimics and luciferase reporters containing ATB-WT or ATB-MUT transcript. **P < 0.01 vs. miR-200a NC group. **d-e** RNA-IP with anti- antibody was performed in U251 and A172 cells transfected with miR-200a NC and miR-200a mimics. ATB expression level was detected using RT-QPCR. **P < 0.01 vs. miR-200a NC group. Data were presented as mean ± SD from three independent experiments

To validate the direct binding between miR-200a and ATB at endogenous levels, we constructed luciferase reporters, which contain wild-type (WT) or mutated (Mut) miR-200a binding sites (Fig. 4a). We found that overexpression of miR-200a could reduce ATB-WT luciferase activity but not affect ATB-Mut luciferase activity compared with miR-200a NC (Fig. 4b-c). The microRNAs are known to bind their targets and cause translational repression and/or RNA degradation in an Ago2-dependent manner. To determine whether ATB was regulated by miR-200a in such a manner, we conducted anti-Ago2 RIP in U251 and A172 cells transiently overexpressing miR-200a. Endogenous ATB pull-down was specifically enriched in miR-200a-transfected cells (Fig. 4d-e), supporting that miR-200 s are bona fide ATB-targeting microRNAs. These data demonstrated that miR-200a bound to ATB but did not induce the degradation of ATB. All these data implied that ATB physically correlated with the miR-200a in glioma cells.

### Repression of miR-200a restored the sh-ATB induced inhibitory effects on glioma cells

**Fig. 5:**
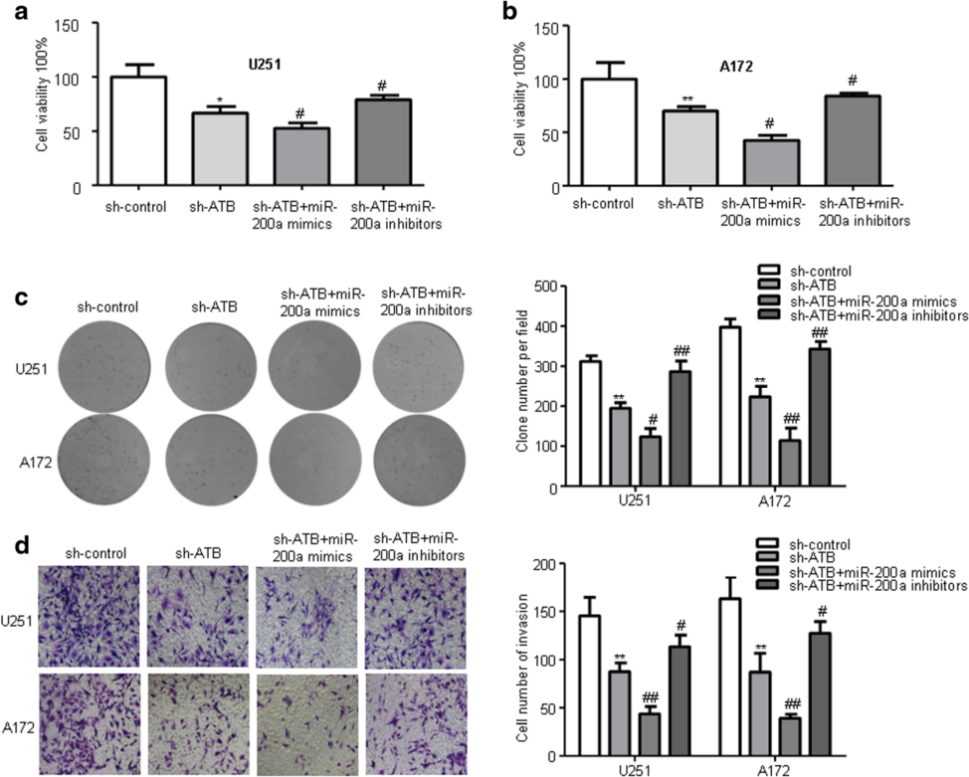
Repression of miR-200a abolished the sh-ATB induced inhibitory effects on glioma cells. **a**-**b** CCK-8 assay was performed to determine the proliferation after co-transfected with sh-ATB and miR-200a mimics or miR-200a inhibitors. *P < 0.05, **P < 0.01 vs. sh-control group; ^#^ *P* < 0.05 vs. sh-ATB group. **c** Colony formation assay was performed to detect the proliferation effect of U251 and A172 cells after co-transfected with sh-ATB and miR-200a mimics or miR-200a inhibitors. *P < 0.05, **P < 0.01 vs. sh-control group; ^#^ *P* < 0.05, ^##^ *P* < 0.01 vs. sh-ATB group. **d** Invasion assay (use matrigel transwell chambers) in U251 and A172 cells was performed to determined cell invasiveness after co-transfected with sh-ATB and miR-200a mimics or miR-200a inhibitors. **P < 0.01 vs. sh-control group; ^#^ *P* < 0.05, ^##^ *P* < 0.01 vs. sh-ATB group. Data are presented as mean ± SD from three independent experiments

In order to investigate whether ATB exerts biological functions through miR-200a, we perform a rescue experiment. The proliferation was reduced in ATB knockdown, while miR-200a inhibitors reversed the reduction of proliferation and miR-200a mimics inhibited the proliferation (Fig. 5a-b). Colony formation assay was used to further assess the proliferation ability. The results showed that ATB knockdown combined with miR-200a overexpression resulted in significant reduction of clone numbers and clone size of glioma cells, whereas miR-200a inhibitors recused the clone ability in ATB knockdown glioma cells (Fig. 5c). In addition, ATB knockdown combined with miR-200a overexpression group was strongly reduced invaded cells, and miR-200a inhibitors reversed the invasion of ATB knockdown glioma cells (Fig. 5d). Therefore, these results suggest that ATB acts its tumor-oncogene roles though miR-200a in glioma cells.

### ATB regulated TGF-β2, a target of miR-200a, in glioma cells

**Fig. 6:**
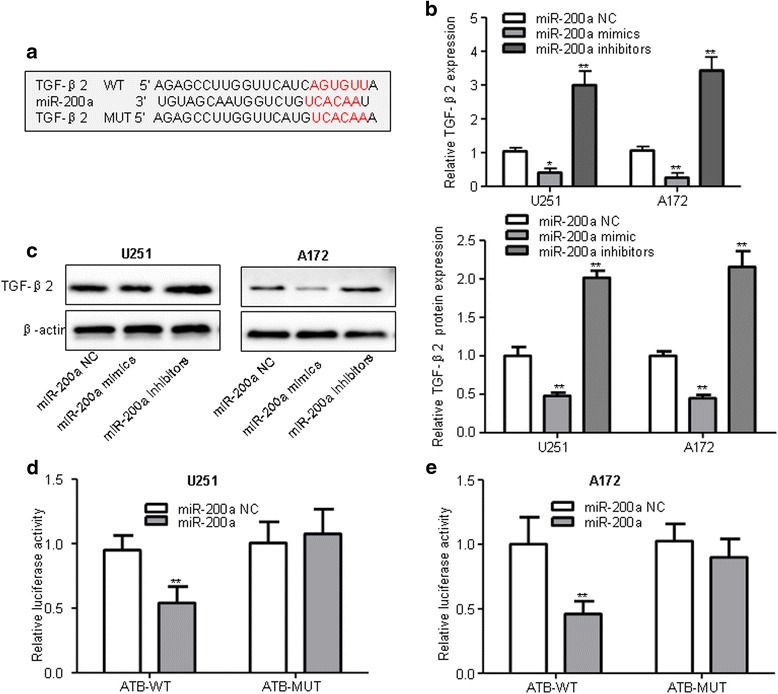
miR-200a directly targeted the 3′UTR of oncogene TGF-β2 and inhibits its expression in glioma cells. **a** The predicted of miR-200a binding sites in the 3′-UTR region of TGF-β2 (TGF-β2-3′-UTR-WT) and the corresponding mutant sequence (TGF-β2-3′UTR-MUT) was shown. **b**-**c** Relative expression of TGF-β2 mRNA and protein levels in U251 and A172 cells after transfected with miR-200a mimics, miR-200a inhibitors, and miR-200a NC. *P < 0.05, **P < 0.01 vs. miR-200a NC group. **d**-**e** Luciferase activity in U251 and A172 cells co-transfected with miR-200a mimics and luciferase reporters containing TGF-β2 wild type (WT) or mutant type (MUT) 3′-UTR. **P < 0.01 vs. miR-200a NC group. Data are presented as mean ± SD from three independent experiments

To investigate whether miR-200a targeted TGF-β2 in glioma cells, and TargetScan software predicted miR-200a binding sites in the 3’UTR of human TGF-β2 (Fig. 6a). The expression levels of TGF-β2 in glioma cells transfected with miR-200a NC, mimics and inhibitors were tested. Overexpression of miR-200a significantly reduced both mRNA and protein expression levels of TGF-β2 compared to miR-200a NC while inhibited miR-200a expression exhibited the opposite effects (Fig. 6b-c). The dual-luciferase reporter assay showed that the luciferase activity in the TGF-β2-WT was significantly decreased after transfection with miR-200a mimics compared to miR-200a NC, whereas the luciferase activity in the TGF-β2-MUT had no change in cells transfected with miR-200a mimics and miR-200a NC group (Fig. 6d-e). These data indicated that miR-200a inhibited TGF-β2 expressions in glioma cells by directly targeting the 3′UTR of oncogene TGF-β2.

**Fig. 7:**
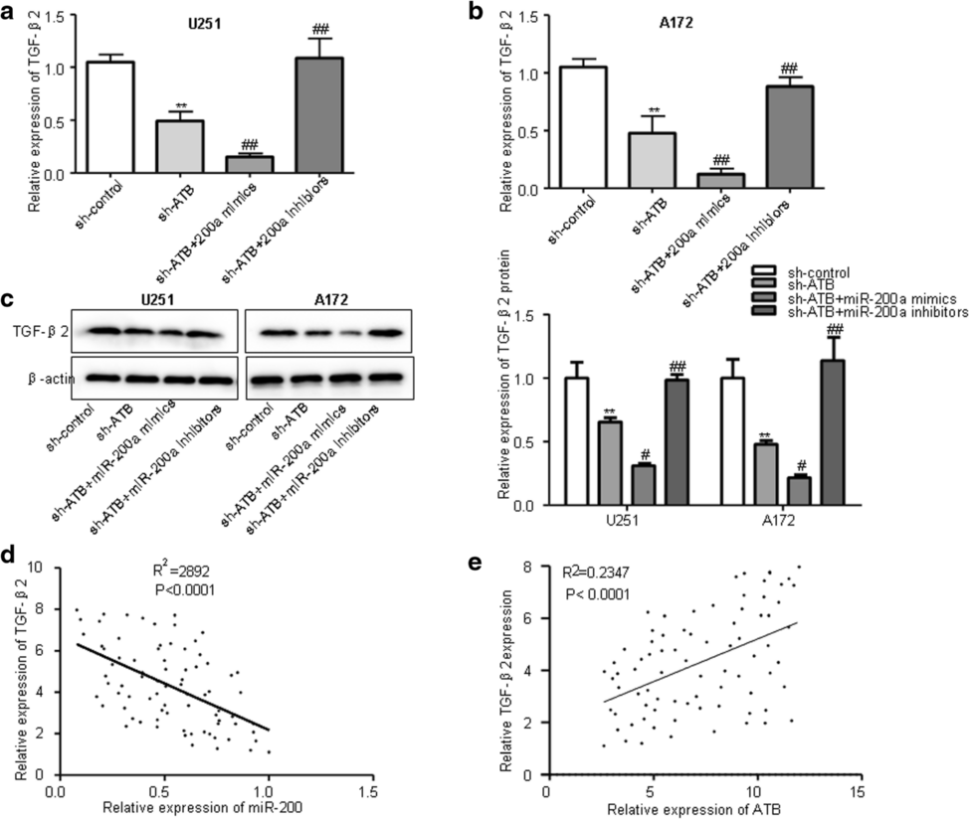
ATB as a ceRNA for TGF-β2 by releasing miR-200a in glioma cells. **a**-**b** RT-QPCR and **c** Western blot assays were performed to detect the mRNA and protein levels of TGF-β2 after cells transfected with sh-ATB and miR-200a mimics or miR-200a inhibitors. **P < 0.01 vs. sh-control group; ^#^ *P* < 0.05, ^##^ *P* < 0.01 vs. sh-ATB group. Data are presented as mean ± SD from three independent experiments. **d** Pearson’s correlation analysis of the relationship between TGF-β2 expression and miR-200a expression. **e** Pearson’s correlation analysis of the relationship between TGF-β2 expression and ATB expression

To explore whether ATB could function as a ceRNA for TGF-β2 via modulating miR-200a in glioma, RT-QPCR and Western blot assays were performed to detect the mRNA and protein levels of TGF-β2 after cells transfected with sh-ATB combined with miR-200a mimics, miR-200a inhibitors. Knockdown of ATB reduced the expression of TGF-β2 at mRNA and protein levels, and sh-ATB combined with miR-200a mimics significantly reduced the expression levels of TGF-β2, in contrast, miR-200a inhibitors restored the reduction of TGF-β2 expression in ATB knockdown glioma cells (Fig. 7a-c). Additionally, we further found that the expression levels of TGF-β2 was inversely correlated with the expression of miR-200a and positively associated with ATB in 79 human glioma patients (Fig. 7d-e). These findings suggest that ATB functions as a ceRNA to regulate the expression of TGF-β2 by releasing miR-200a in glioma cells.

### ATB inhibition significantly suppressed tumor growth in vivo

**Fig. 8:**
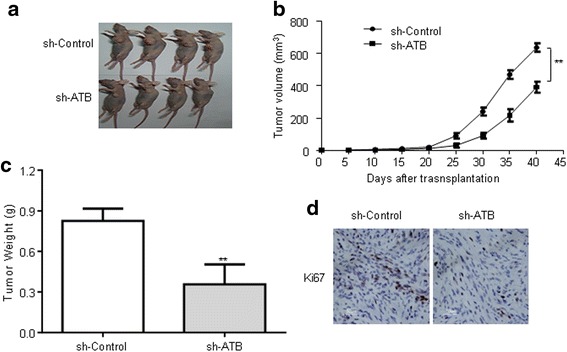
Knockdown of ATB inhibited U251 cell growth in vivo. **a** sh-control or sh-ATB was transfected into U251 cells, which were injected in nude mice, respectively. **b** Tumor volumes were calculated every 5 days after injection. Bars indicate SD. **c** Tumor weights are represented as means of tumor weights ± SD. **d** Immunohistochemical (IHC) staining expression of Ki-67 in subcutaneous tumors of mice injected with sh-control or sh-ATB cells. Data are presented as mean ± s.d. from three independent experiments. **P < 0.01 vs. sh-control group

To evaluate the functional roles of ATB in vivo, sh-control/sh-ATB transfected U251 cells were inoculated into nude mice respectively. Tumor volumes in the sh-ATB group were obviously smaller compared with the sh-control group (Fig. 8a-b). Also, tumor weights in the sh-ATB group were significantly lower than in the sh-control group (Fig. 8c). Ki-67 staining was performed to measure the proliferation ability in xenografted tumor tissues. As shown in Fig. 8d, the sh-ATB group had fewer proliferative cells than that in the sh-control group.

## Discussion

Nowadays, operation and chemoradiotherapy termed as comprehensive treatments are the main means for the treatment of glioma patients, although it can alleviate patients’^,^suffering and prolong life-span, the prognosis is still poor [20, 21]. Therefore, it is urgent for us to find new effective therapies for glioma patients. Recent studies have suggested that dysregulation of lncRNAs are involved in the tumorigenesis and progression of multiple cancers, including glioma [22, 23]. Consequently, understanding the underlying mechanisms and biological functions of lncRNAs in glioma may give us a new direction for the treatment of glioma patients.

A number of studies have demonstrated that lncRNAs play considerable functional roles in human malignant tumor [24–27]. Long non-coding RNA ATB was overexpressed in hepatocellular carcinoma, which promotes HCC cells invasion and tumor growth [13]. Furthermore, ATB is also found as a novel biomarker of lncRNA, indicative of a poor prognosis in gastric cancer and colon cancer patients [15, 28]. In the present study, our results confirmed that ATB was highly expressed in glioma, and high ATB expression was associated with a poor clinical outcome in glioma patients. Knockdown of ATB suppressed the proliferation, migration and invasion of glioma cells. In addition, the in vivo experiments also supported the above findings, suggesting that ATB exerted as an oncogene in human glioma. Moreover, overexpression of ATB in breast cancer cells promotes the invasiveness, which is consistent with the results of our study [14].

MiRNAs may function as oncogenes and/or tumor suppressors in many cellular processes during cancer initiation and progression [29]. Accumulating evidences have showed that lncRNAs can antagonize miRNA function by competing with miRNAs for binding to shared target mRNAs, and then to silence target mRNAs [30]. Long noncoding RNA ANRIL promotes tumor growth by epigenetically repressing of miR-99a/miR-449a in human gastric cancer [31]. Gas5 suppresses the malignant biological characteristics by down-regulating miR-222 in human glioma [32]. Growing number of present work confirmed the common existence of a widespread interaction network of competitive endogenous RNAs (ceRNAs), in which lncRNAs may exert functions through targeting miRNAs and regulating their function role [19, 33]. ATB could promote the invasion-metastasis cascade in HCC by negative regulating of miR-200 family [13]. Previous study showed that down-regulation of miR-200a promoted glioma malignancy by up-regulating SIM2-s [18]. Our present study confirmed that miR-200a was significantly decreased in glioma tissues and inversely correlated with ATB. To explore whether ATB has miR-200a binding site in glioma cells, and was confirmed by luciferase reporter assays and RIP. We verified that ATB is directly bound to miR-200a on ATB transcript. RNA-IP assay showed that the expression of ATB immunoprecipitated with in the miR-200a overexpression group was significantly increased. Meanwhile, we found that ATB exerted the function of anti-tumor by miR-200a in glioma cells. Taken all together, these data strongly suggested that ATB directly targets miR-200a and affects the biological characteristic of glioma cells by negatively regulating miR-200a.

The transforming growth factor-β2 (TGF-β2) belongs to TGF-β family, functioned as an oncogene in several cancer types, which promote cancer cells^,^ malignant behaviors [34]. Recent study has reported that miR-200a suppresses renal cell carcinoma development by directly targeting TGF-β2 [35] and miR-200a prevents renal fibrogenesis by suppressing of TGF-β2 [36]. Consistent with previous studies, we confirmed that miR-200a regulated the expression of TGF-β2 via targeting the 3′UTR of TGF-β2.

Emerging evidences indicated that lncRNAs play a critical role in a variety of cellar biological processes by acting as a ceRNA or a molecular sponge in modulating the role and functions of miRNAs [30, 37]. These lncRNAs act as a natural miRNA sponge to control endogenous miRNAs by using shared miRNAs responsive elements (MREs) and then modulating the derepression of these miRNAs targets via post-transcriptional regulation [38]. For instance, HOTAIR, a well-known lncRNA could inhibit the expression of FGF1 by regulating miR-326 in human glioma [39], and also functioned as a competing endogenous RNA to regulate HER2 expression by sponging miR-331-3p in promoting gastric cancer [40]. To further explore whether TGF-β2 is involved in the ATB acts as a ceRNA in regulating the biological characteristics of glioma by modulating miR-200a. We demonstrated that miR-200a reversed the reduction of TGF-β2 mediated by ATB knockdown. In addition, we found that TGF-β2 expression was negatively correlated with miR-200a, but positively associated with ATB in glioma tissues. These results suggested that ATB functions as a ceRNA via decreasing miR-200a, up-regulating TGF-β2 in human glioma.

## Conclusion

In summary, we first reported that ATB was highly expressed in glioma tissues and acted as an oncogene, which serves a key function in regulating glioma malignancy. ATB knockdown suppressed glioma biological characteristics by directly targeting miR-200a and negatively regulating its expression in glioma cells. TGF-β2, a target oncogene, is directly bound to tumor-suppressor gene miR-200a and is involved in ATB function as a ceRNA for miR-200a in glioma. Therefore, understanding the functional role of ATB in glioma will provide us a novel strategy to find potential therapeutic targets for the treatment of glioma.

## Abbreviations

ceRNAs, competing endogenous RNAs; CRNDE, Colorectal neoplasia differentially expressed; DMEM, Dulbecco’s modified Eagle’s medium; FBS, fetal bovine serum; HCC, Hepatocellular carcinoma; HOTAIR, HOX transcript antisense intergenic RNA; LncRNA-ATB, long noncoding RNA activated by TGF-β; lncRNAs, long non-coding RNAs; MEG3, Maternally Expressed Gene 3; miRNAs, microRNAs; MREs, miRNAs responsive elements; NBTs, normal brain tissues; ncRNAs, non-coding RNAs; RIP, RNA immunoprecipitation; TGF-β2, transforming growth factor-β 2
